# Analysis of the Effect of Exams on Perceived Stress and Temporomandibular Joint Status in Physiotherapy Students: A Pilot Study

**DOI:** 10.3390/medicina60060952

**Published:** 2024-06-07

**Authors:** Marta Macarena Paz-Cortés, Andrea Martín-Vacas, Pedro Antonio Mendoza, Manuel Rozalén, Miguel Ángel Alvaredo

**Affiliations:** 1 Faculty of Dentistry, Alfonso X El Sabio University, 28691 Villanueva de la Cañada, Spain; mpazcor@uax.es (M.M.P.-C.);; 2 Physiotherapy Department, Faculty of Health Sciences, Alfonso X El Sabio University, 28691 Villanueva de la Cañada, Spain; pmendmor@uax.es (P.A.M.); mbustroz@uax.es (M.R.)

**Keywords:** temporomandibular joint, temporomandibular disorder, stress, students, physiotherapy

## Abstract

*Background and Objectives*: Temporomandibular disorders or dysfunction (TMDs) encompass a range of complex conditions that impact the temporomandibular joint (TMJ), chewing muscles, teeth, and/or their supporting tissues. Stress is one of the most associated factors studied as a TMD predictor. The aim is to figure out the influence of stress on TMJ status and salivary cortisol in university students during and before exams. *Materials and Methods*: The study was non-experimental, employing a longitudinal, analytical, observational cohort design. The study population consisted of students enrolled in the physiotherapy degree program at the Alfonso X El Sabio University (Madrid, Spain). Data were collected during two distinct academic periods: the first period was characterized by low academic stress and no exams, and the second period coincided with the high academic stress of final course exams. The collected results included sociodemographic data, assessment of TMJ status (Fonseca Index), evaluation of muscle evaluation (masseter, upper trapezius, and sternocleidomastoid) using a MOXY Monitor (muscle oxygenation) and Neurotrac^®^ (surface EMG, sEMG), assessment of perceived stress (PSS-14), and measurement of salivary cortisol (enzyme immunoassay with Elisa). The statistical analysis was conducted with a confidence level of 95% (*p* ≤ 0.05) and asymptotic or bilateral significance. *Results*: 70 students were analyzed during two different measurement periods. According to the Fonseca Index, initially, 37.14% of the analyzed students showed mild TMDs, 17.14% moderate TMDs, and 45.72% showed no TMDs. In general terms, stress increased with age and is related to female sex, maximum mouth opening decreased with age, oxygen saturation decreased with age and academic stress, and myoglobin concentration was related to age. Furthermore, muscle contraction decreased during higher academic stress and increased with age. For women, age was a risk factor for suffering from TMDs, and stress worsened the transition from having TMDs to having no TMDs in both sexes. *Conclusions*: Academic stress influences TMJ status and muscle outcomes such as oxygen saturation, myoglobin concentration, and muscle contraction, although more research is needed.

## 1. Introduction

Temporomandibular disorders (TMDs), also known as temporomandibular dysfunction, constitute a multifaceted group of conditions that directly impact the bone structures and soft tissues within the orofacial region. These structures include the temporomandibular joint (TMJ), masticatory muscles, teeth, and their associated support apparatus [1,2]. Most researchers agree that these problems can be divided into three main categories: (1) myofascial pain, discomfort or pain in the muscles related to the jaw, (2) structural asymmetry of one of the joints caused by a displaced disc or injury to the condyle, and (3) arthritis refers to a group of inflammatory and degenerative joint disorders that can affect the TMJ.

Currently, the etiology of TMDs is complex and multifactorial. Researchers have identified various genetic and epigenetic factors that may contribute to the onset and persistence of pain and functional changes associated with TMDs. These include biological, environmental, social, behavioral, and emotional factors, which continue to be a topic of discussion among different authors [3,4,5]. Skeletal malocclusion is described a factor that can influence the limitation of mandibular opening, producing TMJ noises [1,2,6], shown mostly in skeletal retrognathia, although the quality of the available literature does not seem high enough to provide strong scientific evidence on the subject [7]. Another factor associated with TMDs is stress, which causes muscle tension and/or psychosomatic reactions [8]. The levels of emotional stress experienced increase the tone of the muscles of the head, neck, lower back, and shoulders [9,10,11,12,13], and increase levels of non-functional muscle activity. Saccomanno et al. [14] found that the presence of anxiety and stress increased the frequency, intensity, and duration of functional habits (bruxism), overloading the TMJ [15].

Acute psychological stress activates the sympathetic adrenal medullary nervous system (SAM), leading to the release of catecholamines in the adrenal medulla. At this point, the activity of the slower-acting hypothalamic–pituitary–adrenal (HPA) axis increases. In response to SAM activation, the nervous system releases catecholamines (adrenaline and noradrenaline), which stimulate an increase in heart rate and blood pressure [16]. It also reflects an activation of salivary alpha-amylase levels. On the other hand, the endocrine system responds through the activation of the HPA axis, leading to the release of corticosteroids, mainly cortisol in the adrenal cortex, considered one of the main biomarkers of stress [17,18]. Cortisol is a steroid hormone with a catabolic behavior, which interferes with numerous processes such as homeostasis, sleep regulator, and, of course, in the perceived stress that is synthesized in the adrenal glands from cholesterol and whose levels depend on the circadian rhythm. The release of cortisol is beneficial as it has been associated with improving cardiovascular and anti-inflammatory functioning of immune systems, influences glucose metabolism, and affects affective and cognitive function to help the body cope with stress. Due to cortisol’s extensive reach across multiple biological systems, dysregulation in its production poses a risk, increasing the risk of stress-related diseases [18,19,20]. Cortisol fulfills a variety of crucial roles including mediating and suppressing healthy stress responses. However, chronic exposure can lead to structural changes in brain regions responsible for modulating the stress response, such as the hippocampus, and may contribute to the pathophysiology of anxiety and mood disorders [21].

The increase in chewing muscle activity because of stress produces an overload in the TMJ, which can alter functional activity during mastication [9,14,22]. This stress can also influence the individual’s sympathetic activity, producing increased muscle tone and pain, showing a relationship between stress and TMJ pain [23]. In highly stressful situations, there is an increase in cortisol levels [24], triggering a decrease in muscle strength and mass, leading to sarcopenia and even physical alterations of muscle itself [25]. It has been seen that higher values of salivary cortisol are related to alterations in the TMJ [26].

In general, TMDs have a prevalence of approximately 17–26% among the general population, of which 11.7% become chronic [27], and being more common in the female gender. Data from a recent systematic review and meta-analysis indicate that the global prevalence of TMDs has risen to 34%. The age group 18–60 years is the most affected, with women being particularly vulnerable. Furthermore, a higher prevalence is reported in South America (47%) compared to Asia (33%) or Europe (29%) [28]. In a recent study conducted in Switzerland, a prevalence of 31.41% of TMJ alterations in adults was reported, with 24% exhibiting flattening, 2.75% deformation, and 4.67% indeterminable conditions [29]. Another study in a Croatian sample established that students of biomedical careers have an increased risk for TMDs related to their lifestyle habits [30]. It is important to consider that during the COVID-19 pandemic, the deterioration of the population’s psychological state increased, worsening psychogenic diseases, and increasing the severity of chronic facial pain [31]. Moreover, an increase in the prevalence and symptomatology of TMDs was observed, with up to 40.7% of participants exhibiting symptoms of TMD in the previous month [14]. In addition, according to a recent systematic review and meta-analysis, patients with TMDs reported moderate-to-severe distress and stress reactivity [32], and cross-sectional studies showed that women are 1.5 to 2 times more likely to suffer from TMDs than men [27,33,34]. In recent investigations, it was concluded that students who suffer from stress are more likely to suffer changes in muscle asymmetry during the act of clenching their teeth [11], showing an increase in muscle tone of the chewing muscles depending on the stress perceived by the student [15]. The high prevalence of TMDs in Europe positions university students as a particularly vulnerable group due to university stress. For this reason, we believe that it is relevant to conduct a study that evaluates the stress of students before and during exams, in addition to relating it to TMDs with the objective of carrying out prevention methods from universities. The aim is to determine the influence of stress on TMJ status and salivary cortisol in university students during and before exams.

## 2. Materials and Methods

The study was non-experimental, employing a longitudinal, analytical, observational cohort design.

### 2.1. Population and Sample Selection

The study population consisted of students enrolled in the physiotherapy degree program at Alfonso X El Sabio University (UAX) in Villanueva de la Cañada, Madrid. The inclusion criteria required participants to be from the second course (1), enrolled in the “Special Physiotherapy I” subject (2), and willing to take part in the study (3). Exclusion criteria included individuals with facial hair (e.g., beard) (1), those who had undergone TMJ surgical treatments (2), or were taking stress medication (3) to prevent electrode measurement errors. None of the researchers taught classes in the subject, avoiding potential biases due to personal affinity to researcher during measurements. The criteria for sample selection were similar to those used in previous comparable studies [14,35].

All students enrolled in the subject were invited to participate, and ultimately, only those who voluntarily expressed interest in being part of the study and signed the informed consent form were included. The sampling method used was non-probabilistic, consisting of consecutive cases until the predetermined sample size was reached. The sample size was determined using the “one in ten” rule [35,36,37,38]; in this approach, the number of subjects is calculated based on the number of variables in the study plus a constant, which establishes a ratio of ten subjects for each variable. It is important to note that if the variable is quantitative, ten subjects are added per variable, and if it is categorical, ten subjects are added for each category except one [39]. In the present study, a total of nine variables were analyzed (stress, cortisol, maximum mouth opening, oxygen saturation, muscle contraction, age, sex, myoglobin concentration, and measurement period), so a total of nine parameters plus the constant; a total of 100 study subjects would be required to meet the premise.

$$n=\left(9\times 10\right)+10=100$$

Finally, 70 students participated in the study, with a prevalence of TMD of 54.29%, obtaining a margin of error of 6.42% in detecting pathology with a confidence level of 95%, so we considered that the sample collected met quality criteria and representativeness.

### 2.2. Ethical Aspects

The information collected related to this study was strictly necessary for the specific research objectives and was processed in compliance with the General Regulation (EU) 2016/679 and Organic Law 3/2018, on protection of personal data and guarantee of digital rights. The university is responsible for the treatment and custody of the participants. An informed consent form was provided to explain the nature of the study, which participants were required to sign to participate. Each subject was assigned an identification number to preserve their anonymity. Additionally, the study adhered to the principles of the Declaration of Helsinki, modified at the 64th General Assembly, Fortaleza, Brazil, October 2013. Approval for the study was obtained from the ethics committee of the “Clínico San Carlos” Hospital in Madrid, Spain (code H 22/682-E).

### 2.3. Variables and Measurements

A data collection notebook was created to compile the variables measured during each diagnostic test and gather sociodemographic data. All measurements were conducted on the same day, and both the instruments and their usage validated prior to the study. The initial measurement was carried out during the non-exam period at the beginning of the semester (13 March 2023), and the second measurement was conducted during the exam period at the start of the final exams (15 May 2023). Despite the ongoing alert for the COVID-19 pandemic in Spain, the Council of Ministers agreed to declare the end of the health crisis situation in Spain, as reflected in Order SND/726/2023 published in the BOE on 5 July 2023. Changes between the first and the second measurement period were analyzed. Samples were collected at the Physiology Laboratory of the Virtual Hospital of the UAX (Villanueva de la Cañada, Madrid, Spain).

1. Sociodemographic data: Date of birth and date of measurement were recorded to calculate age in years (continuous quantitative). Additionally, sex was also documented (dichotomous qualitative).
2. Evaluation of TMJ status: To assess TMDs, a diagnostic method was employed, performed by the same dentist (M.M.P.-C.), calibrated in previous research, and an expert in orofacial pain and TMDs, to mitigate operator bias. The Fonseca Anamnestic Index, a verbal questionnaire directed by a dentist, was utilized. This questionnaire comprises ten questions, with response options categorized as: no (0), sometimes (5), or yes (10). The summation of the responses categorizes TMJ disorders into no disorder (0–15), mild disorder (20–40), moderate disorder (45–65), and severe disorder (70–100) [40]. Opening, laterality, protrusive movements, pain on palpation, and pain on movement were assessed according to the criteria outlined in the Helkimo index [41] modified by Maglione in 1986 [42]. Prior to the dental assessment, the students used PHB 0.12% CHX (PHB) mouthwash to prevent cross-infections. Measurements were carried out using an electronic caliper (Vietnam E-Commerce Limited, RM 18 27/F, Ho King, COMM CTR 2 16 170 FA Yueng St. Mongkok, Kowloon, Hong Kong).
3. Muscle assessment: for muscle evaluation, two devices were utilized. Neurotrac^®^ 4 Myoplus (Neurotrac, Southampton, UK) is a surface electromyograph, and MOXY Monitor (Fortiori Design LLC, Hutchinson, MN, USA) is a non-invasive monitor designed to measure muscle oxygen saturation. The technical specifications of the electromyograph include dual-channel EMG functionality, with an EMG range of 0.2–2000 μV RMS (extended), a sensitivity of 0.1 μV RMS, and an accuracy of 4% of μV ± 0.3 μV indications at 200 Hz. Regarding the selective band filter of 3 dB, it can be either wide (18 Hz ± 4 Hz to 370 Hz ± 10%—reading below 235 microvolts 10 Hz ± 3 Hz to 370 Hz ± 10%—reading above 235 microvolts) or narrow (100 Hz ± 5% to 370 Hz ± 10% 1.5 Notch filter: 50 Hz–33 dbs 0.1% accuracy) [43]. The measurements were performed by the same physiotherapist (M.A.A.) to mitigate operator bias. The recorded measures were muscle contraction (mV), oxygen saturation (%), and myoglobin concentration (g/dL). According to the specifications outlined in ANSI/AAMIEC12:2000 for the electrodes utilized, in accordance with the manufacturer’s instructions, the average impedance value measured at 10 Hz for 12 pairs of electrodes is reported at 95 Ω (with a maximum ANSI/AAMI value of 2 kΩ). Additionally, the impedance at 10 Hz after testing is recorded as 77 kΩ (with a maximum ANSI/AAMI value of 3 kΩ) [44]. For the electrode placement, the skin was first cleaned, and then circular self-adhesive electrodes (25 mm, Tens Care) were applied using a 10 cm × 10 cm dressing (Hypafix). A silicone biter was inserted between the molars to perform maximum voluntary contraction (MVC). Following this, simultaneous data collection from *Neurotrac***^®^** and *MOXY Monitor* were obtained, and three isometric MVCs of three seconds and the average of the recorded data were calculated, leaving five seconds between each MVC [45,46]. For the measurements, the subjects were placed in a seated position, following Delcanho et al. [45]. The situation of the electrodes for sEMG in each muscle was:
  - Masseter muscle: For the sEMG, we followed the references outlined by Delcanho et al. [45], which specified a point located 20 mm from the angle of the mandible, along a line connecting the angle with the wing of the nose. Two Neurotrac^®^ sensors were placed on each side, along with one on each side of the *MOXY Monitor*.
  - Upper trapezius or trapezius descendens: For sEMG, the electrodes were positioned midway between the acromion and seventh cervical vertebra, and the distance between them is 20 mm, as outlined in SENIAM guidelines [47]. For the *MOXY Monitor*, it was necessary to place the electrodes at the cross-section of the muscle, halfway between the acromion and C7. The distance between these electrodes was set at 30 mm [48].
  - Sternocleidomastoid (SCM): For sEMG, the electrodes were placed between the mastoid process and the medial third of the clavicle, maintaining a distance of 10 mm between them. For the *MOXY Monitor*, the placement coincided with the placement of the electrodes in the *Neurotrac^®^.* The distance between the electrodes was standardized at 25 mm for the SCM [48].
4. Measurement of perceived stress: A questionnaire, the Perceived Stress Scale (PSS-14), was administered in person, by a psychologist (M.A.B) to mitigate operator bias. The PSS-14 [49] is a scale comprising 14 questions designed to assess the perception of stress over the past month. Each question is associated with a specific value based on the response category: never (0), almost never (1), occasionally (2), often (3), and very often (4). The total score is interpreted as follows: scores ranging from 0 to 14 indicate never or almost never experiencing stress; 15–28, occasionally stressed; 29–42, frequently stressed; and 43–56, very frequently stressed.
5. Salivary cortisol: A salivary sample collection was conducted by a nurse (V.O.), experienced in oral sampling, to mitigate operator bias. Participants were instructed not to eat or smoke 60 min before sample collection, to rinse with water, and to wait at least 10 min before collection to prevent the sample from being diluted. Saliva sampling was always conducted in the morning, between 8:00 am and 12:00 am. A volume of 2 mL of unstimulated saliva was collected over a period of 4 min, using a passive technique. Salivette^®^ kits (Sarstedt AG & Co. KG, 51588 Nümbrecht, Germany) were utilized for saliva collection, and the analysis method employed was enzyme-linked immunoassay (ELISA), with reference values in the morning ranging from 1.19 to 7.21 ng/mL. Samples were analyzed at a clinical analysis laboratory in Madrid (ABOLAB, Madrid, Spain). Throughout the day of collection, the samples were stored in refrigerated medium (Refrigerator ATEX FPX 50 ATEX) and transported to the clinical laboratory on the same day for analysis. The morning cortisol pickup was chosen due to its peak release upon waking and subsequent gradual decrease throughout the day. Diurnal cortisol activity is subject to the influence of the individual chronotype, with morning or evening preferences impacting various physiological systems and parameters such as catecholamine secretion [50,51,52,53,54].

### 2.4. Statistical Analysis

The statistical analysis of the data was conducted in two stages using the statistical software Stata 16.0 (Stata Corp, College Station, TX, USA). The tests were performed with a confidence level of 95% (*p* ≤ 0.05) and asymptotic or bilateral significance. First, a descriptive analysis of the data was carried out. Depending on the variable type, different values were reported: for quantitative variables, the mean, standard deviation, maximum, and minimum were calculated, while for categorical variables, absolute and relative frequency and percentages were reported. Secondly, an inferential analysis of the data was conducted. Ordinal logistic regression and multiple linear regression techniques were used to analyze differences between TMJ status, stress levels, muscle condition, and salivary cortisol level during a period with exams (May) and another without exams (March). Additionally, the statistical models were adjusted for sex, age, course of study, and number of years studying the degree.

## 3. Results

### 3.1. Sample Description

A total of 70 students (38 male and 32 female) from the physiotherapy degree program at “Alfonso X El Sabio” University (Spain) were analyzed. The mean age was 21.95 years (SD = 3.69) for males and 21.55 years (SD = 2.26) for females (Table 1). Although the number of students who agreed to participate and met the inclusion and exclusion criteria was lower than the calculated sample size (100), with a confidence level of 95% and a proportion of 54.29% of subjects diagnosed with TMDs in the first measurement in March, the margin of error in detecting subjects with TMDs is considered to be 6.42%.

Descriptive statistics of quantitative variables were conducted (Table 2). In March (first measurement period), the mean cortisol concentration was 6.37 ng/mL, and the average stress score on the PSS-14 questionnaire was 30.51 points. The mean oxygen concentration measurements were similar across the three studied muscles, with the standard deviation (SD) of the masseter measurement being higher than those of the upper trapezius and SCM. Regarding myoglobin and both right and left laterality movements, there was not much difference between the muscles in terms of mean and SD. According to the Shapiro–Wilk test for normality, only two variables (MMO and R-Lat) followed a normal distribution. Related to the homogeneity of the variances by sex, variables associated to mandibular movements, the concentration of hemoglobin in the three studied muscles, and the muscle contraction of the masseter and SCM showed homogeneity criteria. In the comparison between the two measurement periods, the mean cortisol concentration decreased by 0.98 ng/mL from the first measurement to the second. However, stress measurement using the PSS-14 questionnaire increased by 1.66 points from March to May. Muscle oxygen saturation values decreased by more than three points in the three muscle groups from March to May. Additionally, muscle contraction in microvolts decreased in May compared to March. Laterality and maximum protrusion movements increased in May by almost 1 mm on average.

**Figure 1:**
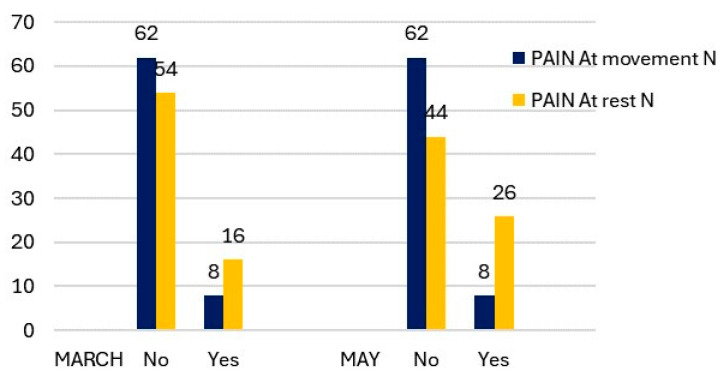
Description of TMJ pain at movement and at rest by measurement period.

**Figure 2:**
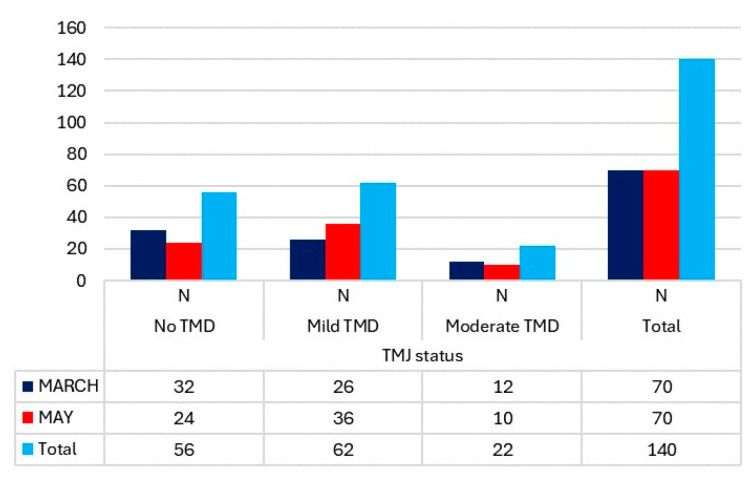
TMJ status, according to the Fonseca Index and differentiated by measurement period, in the students.

The number of cases of TMJ pain on movement and at rest in March showed a significant difference (Figure 1). Only 11.43% of students experienced pain at mandibular movement, while 22.86% reported pain at rest. In the evaluation of the TMJ status using the Fonseca Index, there were no cases of severe TMDs, and only 17.14% of students exhibited moderate alterations. (Figure 2). It is noteworthy that the cases of moderate TMDs decreased by two from March to May. However, there were more cases of low and moderate TMDs in May compared to March (46 versus 38).

### 3.2. Regression Analysis in Total Sample

A multiple linear regression analysis was performed for the quantitative outcomes (Table 3) to examine the relationships with sex, measurement period, and age.

In relation to stress, no significant differences were found. However, when analyzing cortisol levels, significant differences were observed between the measurement periods, with a decrease of 0.98 ng/mL of cortisol from the first measurement period (March) to the second (May). For maximum opening, sex and age were significant variables: it decreased by 5.46 mm in women and decreased by 0.90 mm for each additional year of age (*p* < 0.05 in both comparisons).

Regarding the oxygenation of the masseter, SCM, and upper trapezius, there was a significant relationship with respect to the measurement period, showing a notable decrease in May. Specifically, oxygenation decreased by 3.43% in the masseter, 3.16% in the SCM, and 3.47% in the trapezius (*p* < 0.05 for all). Additionally, female condition increased oxygen saturation in all three muscles: 4.96% in the masseter, 3% in the SCM, and 3.38% in the trapezius (*p* < 0.05 in all). Regarding age, each additional year was associated with a 0.58% decrease in oxygen saturation in the masseter.

Myoglobin concentration decreased in women by 0.17 g/dL in the SCM and 0.23 g/dL in the upper trapezius (*p* < 0.05 in both comparisons). The level of muscle contraction in the masseter was influenced by the measurement period, being up to 76.18 µv higher in May than in March; furthermore, for each additional year of age, muscle contraction increased by 5.02 µv (*p* < 0.05 in both). For the SCM, only gender had a significant effect, with muscle contraction decreasing by 27.36 µv in women compared to men (*p* < 0.001). In the upper trapezius, both the measurement period and gender influenced significantly muscle contraction. During the exam period (May), muscle contraction increased by 98.08 µv. Conversely, women experienced a decrease of up to 44.1 µv in muscle contraction compared to men (*p* < 0.05 in both).

Females had a significantly higher risk of suffering from a moderate TMD compared to not having a TMD, with the risk being multiplied by 17.69 times (*p* = 0.000). Furthermore, for each additional year of age, this risk increased by a factor of 1.19 (*p* = 0.028). In the comparison of having a mild disorder versus not having it, no significant values were found, but there were positive trends favoring the first measurement period (March), being female, and older age, with the risk multiplying by 1.96–2.16 and 0.87 times, respectively (Table 4). Moreover, being female tended to imply a reduction in stress (*p* = 0.063) (Table 5). In the relationship between stress and TMJ status, it was observed that stress increased by 2.34 points when moving from not having TMDs to having mild TMDs (*p* = 0.047). Similarly, stress increased by 7.20 points when moving from not having TMDs to having moderate TMDs (*p* = 0.000).

### 3.3. Regression Analysis in in the Sample Differentiated by Sex

An analysis was conducted differentiating between male and female students, as most of the variables did not exhibit a homogeneous distribution by sex (Table 6).

In relation to stress, for every year that women completed, their stress increased by 1.05 points (*p* = 0.003). In men, although not significant, there is a trend regarding the measurement period, with stress increasing in the first period (May) (*p* = 0.065), and with age, stress decreases for each year completed (*p* = 0.076). Regarding cortisol and sex, there was no significant relationship or trend observed. However, age seemed to significantly influence the ability to open the mouth less, with up to 0.69 mm less in men and a 1.57 mm reduction in women, for each year completed (*p* < 0.05 for both).

In reference to oxygen saturation in the masseter, it decreased by 0.74% for each additional year reached in men (*p* = 0.033), while in women, it decreased by up to 7.24% in the May measurement compared to March (*p* = 0.000). Similarly, the saturation of the trapezius in women was observed to decreased by 6.26% in May (*p* = 0.000). Regarding the distribution of the myoglobin concentration in men, it was observed to increase by 0.02 g/dL for each year reached in both the SCM and the upper trapezius (*p* < 0.05 for both).

Finally, regarding muscle contractions, both in men and women, measuring in May compared to March significantly influenced the masseter and upper trapezius, and in women in the SCM (*p* = 0.000 for all). Age influences the upper trapezius in both sexes, decreasing by 10.81 µv in women and increasing by 9.29 µv in men for each year reached (*p* < 0.05 for both). In the masseter muscle in men, age also has an impact on contraction, increasing by 5.55 µv for each year of age (*p* = 0.043).

In the sex differentiation for analyzing TMJ status, only women’s age is associated with an increased risk transitioning from moderate disorder to having no TMDs (RR = 3.48, *p* = 0.003) (Table 7). In the distribution by sex, in men, stress significantly increased between having a mild TMD (4.37 points) and a moderate one (10.39 points), to having no disorder (*p* = 0.003 and *p* = 0.001, respectively). In women, there is a tendency for stress to increase with a moderate TMDs rather than not having it (*p* = 0.006) (Table 8).

## 4. Discussion

### 4.1. Discussion of the Method

Despite inviting 100 students to participate, those who attended the first measurement but were unreachable for the second measurement were therefore excluded from the study. A total of 70 physiotherapy students from Alfonso X El Sabio University (Madrid, Spain) with an average age of 21.95 years were analyzed. There are few studies in the literature that analyze TMJ status in university students. Although no study precisely mirrors our methodology, we found a similar one conducted exclusively on women (non-university students) [11]. The study conducted is non-experimental, employing a longitudinal analytical observational cohort design, similar to other studies [55,56]. However, in the literature, cross-sectional studies [57,58,59,60], analytical studies of paired samples [61], or case-controls [62] are also observed.

Regarding the sample size, there is disparity in the literature, with studies found having smaller [11,59,61,62,63], similar [64], or larger samples than ours [15,55,56,57,58,60,65]. In terms of age, studies have been conducted in patients under 14 years of age [62,64], ages between 10 and 18 years [59,61], 15 and 30 years [56,58], or 18 and 30 years [55,57,63]. Studies with average ages of 22 years, like ours, are relatively rare, with only three identified [11,15,65]. Regarding the assessment method used, we employed the TMD assessment method described by Fonseca, similar to other studies [58,65,66]. However, other investigations used the Diagnostic Research Criteria for Temporomandibular Disorders (RDC/TMDs) [11,56,57,60,61,62,63,64,67,68]. In relation to the assessment of stress, for the adult population, among the instruments used, the following can be found: the Lipp’s Stress Symptom Inventory for Adults (ISSL) [59,61], Psychological Questionnaire DS14 (type-D scale) [55], Generalized Anxiety Disorder Scale-7 (GAD-7) [68], State-Trait Anxiety Inventory (STAI) [60], and the Perceived Stress Scale PSS-10 by Cohen, Kamarch, and Mermelstei [11,67,69], like ours but more current than the PSS-14.

Regarding the salivary cortisol, the literature indicates that in healthy individuals, cortisol levels peak after the onset of an acute stressor and then return to baseline concentrations as it is eliminated from circulation, with a half-life of 60–70 min [70]. Cortisol is a marker of high specificity in chronic pain compared to other hormones, making it the primary biomarker of psychosocial stress. It has been observed that cortisol can be obtained by venipuncture, but the method of obtaining it itself can lead to an increase in this hormone. Therefore, salivary analysis, despite having the second-lowest concentration of cortisol, has been identified as an ideal method for research studies [21].

Related to the material used to perform the sEMG, various devices are referenced in the scientific literature. Notable examples include the Noraxon Clinical DTS [15,67], Electromyography Miotool (MIOTEC, Porto Alegre, Brazil) [61], and EMG BioEMG III BioPAK Measurement System (BioResearch Associates, Inc., Milwaukee, WI, USA) [11,69,71], among others. Additionally, NIRS (Portamon, Artinis Medical Systems, Elst, The Netherlands) was utilized by Puel et al. [59] to obtain peripheral muscle oxygenation data, with maximal voluntary contraction of the masseter performed through sustained dental occlusion for 20 s.

### 4.2. Prevalence of Temporomandibular Disorders (TMDs)

Related to the prevalence of TMDs, our study was conducted in a sample of university students of physiotherapy. Among the study sample, 45.72% were free of TMDs, 37.14% were diagnosed with mild TMDs, 17.14% were diagnosed with moderate TMDs, and none of our students showed severe TMDs. In accordance, in a similar study [58] employing Fonseca’s method, it was found that 69.4% of their subjects had no TMDs, while 26.6% had mild TMDs, 3.4% had moderate TMDs, and 0.6% had severe TMDs. An earlier study [65] showed similar results, finding 53.2% of students free of TMDs, 36.1% with mild TMDs, 9.6% with moderate TMDs, and 1.1% with severe TMDs. Nomura et al. reported a prevalence of 53.21% of some level of TMDs, divided into 35.78% with mild TMDs, 11.93% with moderate TMDs, and 5.5% with severe TMDs [66].

Other studies conducted on students with different classifications have found varying prevalence rates of signs and symptoms of TMDs. For instance, one study reported a prevalence of 70.6% in students [56]. Wieckiewicz et al. found symptoms in 54% of the students, with disc displacement symptoms being more common (32%) [57]. Similarly, another study [69] reported that 54% of students did not present signs of TMDs, while 46% did, often associated with stress. Studies conducted on students have identified myofascial pain in 66% of cases, myofascial pain and disc displacement in 25%, and disc displacement in 8.3% [61]. Puel et al. found muscular pathology in 73.6% of students, with 26.4% showing a combined condition of muscular diagnosis associated with intra-articular disorders. Among their findings, 63.8% of students did not present TMDs, while 36.2% showed signs and symptoms of these disorders, including disc displacement (42.1%); arthralgia, osteoarthritis, and/or osteoarthrosis (42.1%); muscle disorders with disc displacement (5.2%); muscle disorders with arthralgia, osteoarthritis, and/or osteoarthrosis (2.6%); and disc displacement with arthralgia (7.8%) [59]. In other studies, the overall prevalence of TMDs was reported as 31.7%, with specific prevalence rates for pain-related TMDs, intra-articular TMDs, and combined TMDs being 15.5%, 66.9%, and 17.6%, respectively [60,68]. Following a similar classification in a population of children and adolescents, Al-Khotani et al. found that 72.8% of their sample did not present TMDs, 5.7% presented painless TMDs, and 21.5% presented painful TMDs [72]. In childhood populations, results indicated that 43.14% did not present dysfunction, while 23.53% did present disc displacement, and 27.45% had muscle disorders [62]. Regarding sex differences, females tend to present more moderate TMDs (12.86%) than males (2.86%). Conversely, males exhibit a higher number of non-disorders (28.57%) compared to females (11.43%), consistent with findings from other studies [57,62,65,66,72]. However, some studies do not find a clear predilection by sex [68]. Our study is notable for its examination of university students during two distinct periods of academic stress (a period without exams and a period encompassing final annual exams). For this reason, we have conducted a discussion with those of similar studies [15,56,57,60,65,66,67] to standardize the results and establish a definitive conclusion regarding the signs and symptoms of TMJ among students in correlation with their stress levels.

Regarding joint pain in the TMJ, Owczarek et al. observed TMJ pain in 26% of physiotherapy students, contrasting with an 8% prevalence among dentistry students [15]. Meanwhile, Al-Khotani et al. found that 35.7% of males and 64.3% of females with TMDs experienced pain [72]. Furthermore, it has been detected that students may present TMJ pain regardless of their stress levels, with prevalence rates of 33%, 24%, and 25% for low, medium, and high stress, respectively [67], consistent with findings from other studies [69] reporting pain during activities such as mouth opening, chewing, or yawning among 32% of university students. Owczarek et al. analyzed whether there were differences between first- and fifth-year dentistry students, detecting differences in the perception of pain; while only 8% of first-year dentistry students perceived pain in the TMJ, this value increased to 39% in the fifth-year students [67]. In our sample, in accordance with the results of the previous literature, we found 30% TMJ pain at rest and 11.43% during movement.

### 4.3. Relationship between Stress and Temporomandibular Disorders (TMDs)

TMDs have a multifactorial etiopathogenesis, while several authors emphasize the influence of local factors in their development, others emphasize that of systemic factors. Scientifically, the importance of psychological factors, such as high physical–emotional activity and stress, has also been described as an essential role in the etiopathogenesis of TMDs and oral parafunctions [8,14,15,23,57]. Stress is also a crucial factor contributing to TMD development, being defined as one of the factors in the development and exacerbation of TMDs, especially in young people in late adolescence and early adulthood. In earlier studies, it has been possible to determine the relationship between stress and TMDs [9,14,22,57,59], and depression [71]. In previous studies, it has also been possible to figure out the relationship between stress and depression and anxiety with TMDs [64,65,68,72]. However, other studies have found no relationship between anxiety and TMDs [60]. Owczarek et al. conducted a study in first- and fifth-year dentistry students, with significant differences when relating TMDs with stress when using EEP-10 questionnaire [67]. Based on our findings, we noted a significant increase in stress between the two measurements, with a difference of 31.34 points in the PSS-14 questionnaire. Interestingly, age appears to be a predictive factor for stress in women, with stress levels increasing by 1.05 points for every year completed. Although not statistically significant, there is a noticeable trend among men regarding stress levels increasing in the first period (May) and decreasing with age with each year completed.

In relation to stress, the determination of salivary cortisol is important. Da Silva Andrade observed a significant increase in cortisol in women with TMDs compared to the control group (2.890 ± 0.411 mg/dL vs. 2.470 ± 0.486 mg/dL), highlighting the presence of higher values in women than in men [73]. Kobayashi et al. analyzed the influence of the final master’s thesis on salivary cortisol, without finding differences between the TMD group (90.22 μg/dL/min) and the control group (94.21 μg/dL/min) [67]. Nilsson and Dahlström found no significant differences in the mean cortisol value obtained between groups [63]. The observed values were 10.53 nmol/L for the muscle pathology group, 12.61 nmol/L in the articular disc pathology group, and 13.68 (9.96) nmol/L in the control group. Our results indicated that the mean cortisol level was consistent across intra-group comparisons by sex and age, with an average value of 5.88 ng/mL. Notably, significant differences emerged between the measurement periods, revealing a surprising decrease of 0.98 ng/mL in salivary cortisol during the period associated with higher stress (final exams) compared to the period of lower stress. We speculate that this discrepancy may be attributed to cortisol’s role as a biomarker of acute stress, whereas the exams serve as a sustained stressor over approximately 2–3 weeks.

### 4.4. Discussion of the Study Outcomes

The analysis of muscle oxygenation and myoglobin are of great relevance in determining muscle stress. Puel et al. [59] evaluated peripheral muscle oxygenation levels and the prevalence of psychological stress in adolescents with and without TMDs. In the TMD group, a greater metabolic demand was found in the hemodynamic variable of oxyhemoglobin, with statistically significant differences with the control group with respect to the masseter muscle at rest and during contraction. There also were differences in total hemoglobin in the upper trapezius muscle, in which groups differed at rest, and in tissue saturation index during muscle contraction. In all variables, the results were higher in the control group compared to the TMD group. According to our results, we found a significant relationship between the oxygen saturation of the three muscles analyzed (masseter, SCM, and upper trapezius) with respect to the measurement period. In the period of greatest stress (May), we found a decrease in muscle oxygen saturation, with a decrease of 3.43%, 3.16%, and 3.47% in the masseter, SCM, and upper trapezius, respectively. Furthermore, in the female sex, an increase in muscle oxygen saturation was found (4.96% in the masseter, 3% in the SCM, and 3.38% in the upper trapezius). On the other hand, age was only found related to a decrease in the percentage of oxygen in the masseter of 0.58% for each year completed. Furthermore, we analyzed myoglobin concentration, finding a decrease in women compared to men of 0.17 g/dL in the SCM and 0.23 g/dL in the upper trapezius.

The sEMG values have been extensively examined in the previous literature. In the masseter muscle, the level of muscle contraction is affected by the measurement period, showing an increase of up to 76.18 µv in May compared to March. Additionally, there is an increment of 5.02 µv for each year completed (*p* < 0.05 in both cases). Conversely, for the SCM, only gender has a significant influence, with women experiencing a decrease in muscle contraction by 27.36 µv compared to men (*p* < 0.001). In the upper trapezius, both the measurement period and gender significantly affect contraction microvolts. Being in an exam period (May) leads to a rise of 98.08 µv in muscle contraction, while women exhibit a reduction of up to 44.1 µv compared to men (*p* < 0.05 in both cases).

Owczarek et al. described mean EMG values in masseter muscle tone of 66.4 μV (64.4 ÷ 68.5 μV) for the right muscle and 67.9 μV (65.5 ÷ 70.0 μV) for the left in physiotherapy students, and 64.8 μV (RM 63.1 ÷ 66.6 for the right muscle and 63.4 ÷ 66.7 μV for the left muscle) in dental students, with significant differences between both groups of students [15]. There was a positive correlation between the left masseter muscle and the level of anxiety, but not in the right. In the physiotherapy students, the values obtained for muscle contraction as a function of stress according to the PSS-10 test were, in the low-stress group, 61.5 μV and 62 μV; in the moderate-stress group, 66.9 μV and 66.3 μV; and in the high-stress group, 65.4 μV and 67.7 μV, for the right and left masseter, respectively. On the other hand, the dental students presented values of 62.8 μV and 65.5 μV in the low-stress group, 65.7 μV and 67.1 μV in the moderate-stress group, and 66.5 μV and 66.8 μV in the high-stress group, for the right and left masseter, respectively. In another study by Owczarez et al., stress and contraction of the masseter in first- and fifth-year dental students were evaluated [67]. The mean values of the right masseter were 64.8 μV and 66.3 μV for the first- and fifth-year students, respectively, and the mean values of the left masseter were 64.8 μV and 66.8 μV for the first- and fifth-year students, respectively. In both cases, a significantly higher value was found for fifth-year students compared to first-year students. They also analyzed the contraction force depending on the stress level, finding greater contraction force with increasing stress in the right masseter (65.4 μV, 66.5 μV, and 67.5 μV for low, medium, and high stress, respectively) and left (65.9 μV, 66.3 μV, and 68.9 μV for low, medium, and high stress, respectively).

The study of Souza et al. evaluated and compared levels of stress, posture impairments, and electrical activity of the masticatory muscles during chewing, in preadolescents and adolescents with and without TMDs [61]. The sEMG acquisition system has four channels for the masseter and temporal muscles’ electrical activity evaluation, the muscle electrical activity data of the right and left masseter and right and left temporal muscles, during the active and inactive period of chewing for both groups. The data show that there was no significant difference for active and inactive period of chewing considering masseter muscles or active period for temporal muscles comparing groups. Preadolescents and adolescents with TMDs showed greater muscle activation during the inactive period of chewing, when compared to the control group (*p* < 0.05).

Stocka et al. evaluated the activity levels of the different muscles in occlusion. The average values obtained in the group of women were 72.25 μV in the anterior temporalis, 99.25 μV in the masseter, 8.7 μV in the SCM, and 16.5 μV in the digastric [69]. In the male group, the values, respectively, for these muscles were 74.39 μV, 120.45 μV, 8.6 μV, and 16.8 μV. With respect to symmetry, significant values were obtained between men and women between the right and left masseters, with the values obtained being 105.79 μV and 127.33 μV for the right and 92.68 and 117.71 μV for the left. When evaluating the synergy between the muscles, they saw significant differences between the anterior temporalis and right masseter (84.92 μV–106.78 μV) and the anterior temporalis together with the left masseter (81.93 μV–89.88 μV) between women and men, respectively. Mean values of masseter activities in the group of low-stress subjects (110.52 μV) were statistically different from the groups with medium (115.23 μV) and high (100.33 μV) perceived stress (*p* < 0.05). In a different study, Stocka et al. evaluated the levels of depression and its association with maximum intercuspation in another population [71]. For the temporal muscles, the values obtained were 81.30 and 83.42 μV; for the masseter group, 78.97 and 85.96 μV; SCM, 71.75 and 64.85; 81.30 and 83.42 for the digastric of 80.72 and 81.67 μV; the union of the right temporal muscle with the right masseter, 59.90 μV and 67.03; and finally the synergy of the left temporal muscle with the left masseter, 64.17 and 66.21 μV, for the groups of absence and presence of depression symptoms, respectively. They observed significant differences in the masseter muscles between the study groups. Zielinski et al. [11] also evaluated the relationship between perceived stress and muscular activity, like Owczarek et al. [15]. Statistical analysis revealed that there were not differences between the groups in the bioelectrical activity of tested muscles under all conditions (rest, clenching, cotton rollers clenching, mouth opening); on the other hand, there were statistically significant effects of group on the absolute value of the asymmetry index during clenching on dental cotton rollers between different groups of stress levels.

### 4.5. Limitations and Strengths

This study faces certain limitations that warrant consideration. For instance, there may be some degree of information bias, as additional variables such as weekly study hours could influence academic outcomes. However, accessing data on exam grades would involve sensitive information subject to data protection regulations. Moreover, since the study involves two identical interview-type questionnaires administered at different times, there is a risk of response bias, with students potentially providing answers similar to those given previously. To mitigate this, each interview session was conducted by a different psychologist to ensure objectivity. Additionally, the loss of 30 study subjects resulted in a failure to meet the calculated sample size, potentially affecting the generalizability of the results. Nonetheless, we believe this does not significantly impact the statistical findings, given that some models still yielded statistically significant results. Expanding the sample size and conducting the study over multiple academic years could enhance the robustness of the statistical outcomes. It is worth noting that the indices utilized (Fonseca and Helkimo) are somewhat dated but were chosen for their widespread recognition and ease of communication among researchers and in disseminating the study findings. Furthermore, the Helkimo index primarily focuses on variables related to jaw movement and lateralities as part of its diagnostic approach.

One of the notable strengths of this research lies in its longitudinal design involving university students across two distinct periods, aiming to explore the relationship between TMJ status and academic stress during university exams. Given the scarcity of comparable literature on this topic and the acknowledging challenges associated with conducting longitudinal studies involving students, as highlighted by Schiffman et al., this study contributes valuable insights into understanding TMJ issues in the context of academic stress [74]; we advocate for further research endeavors aimed at exploring the relationship between TMJ pathology and stress among university students. Such studies hold potential for preventing pathologies or identifying susceptible individuals. Moreover, we intend to expand upon the current study by enlarging the sample size, including students from other disciplines, and incorporating the latest diagnostic techniques. These efforts are crucial for advancing our understanding and enhancing the preventive measures in this domain.

## 5. Conclusions

Academic stress influence TMJ status and muscle outcomes such oxygen saturation, myoglobin concentration, and muscle contraction, although more research is needed. Initially, 37.14% of the analyzed students showed mild TMDs, 17.14% moderate TMDs, and 45.72% no TMDs. Females has a higher risk of developing a TMDs, and in women, age is associated with an increasing risk of suffering moderate TMDs. Sex, age, and stress influence the risk of developing TMDs. Regarding the measurement period and TMDs, no statistically significant relationship was observed.

## Figures and Tables

**Table 1:** Description of the demographic variables (sex and age) of the study sample.

	Frequency	Age
Sex	*N* (%)	x ± SD ^x^
**Male**	38 (54.29)	22.3 ± 3.69
**Female**	32 (45.71)	21.55 ± 2.26
**Total**	70(100)	21.95 ± 3.13

^x^ SD—standard deviation.

**Table 2:** Description of the quantitative outcomes of the study sample by measurement period.

Variable		N	x ± SD ^x^	Shapiro–Wilk Sig. ^#^ (*p*)	Levene Sig. (*p*)
**MARCH**					
**Cortisol**	-	68	6.37 ± 3.03	0.000 *	0.55
**Stress**	-	70	30.51 ± 5.84	0.004 *	0.46
**Mandibular movements**					
**Maximum mouth opening**	MMO	70	49.87 ± 7.85	0.700	0.79
**Right laterality**	R-Lat	70	8.74 ± 3.04	0.900	0.25
**Left laterality**	L-Lat	70	8.60 ± 2.82	0.013 *	0.37
**Maximum mandibular protrusion**	MMP	70	7.11 ± 2.77	0.017 *	0.62
**Oxygen saturation (SmO_2_)**					
**Masseter**	SmO_2_-M	70	58.07 ± 10.12	0.000 *	0.00 *
**SCM**	SmO_2_-SCM	70	58.82 ± 7.23	0.001 *	0.00 *
**Upper trapezius**	SmO_2_-UT	70	58.31 ± 6.07	0.019 *	0.00 *
**Hemoglobin concentration (ThB)**					
**Masseter**	ThB-M	70	12.33 ± 0.42	0.000 *	0.09
**SCM**	ThB-SCM	70	12.40 ± 0.31	0.012 *	0.85
**Upper trapezius**	ThB-UT	70	12.42 ± 0.28	0.019 *	0.81
**Muscle contraction (Cont)**					
**Masseter**	Cont-M	70	120.08 ± 45.93	0.034 *	0.99
**SCM**	Cont-ECOM	70	58.82 ± 37.31	0.000 *	0.92
**Upper trapezius**	Cont-UT	70	90.92 ± 78.55	0.000 *	0.01 *
**MAY**					
**Cortisol**	-	68	5.40 ± 2.56	0.000 *	
**Stress**	-	70	32.17 ± 7.10	0.005 *	
**Mandibular movements**					
**Maximum mouth opening**	MMO	70	49.86 ± 8.37	0.105	
**Right laterality**	R-Lat	70	9.58 ± 2.60	0.405	
**Left laterality**	L-Lat	70	9.6 ± 2.89	0.144	
**Maximum mandibular protrusion**	MMP	70	8.14 ± 2.43	0.003 *	
**Oxygen saturation (SmO_2_)**					
**Masseter**	SmO_2_-M	70	54.54 ± 8.78	0.000 *	
**SCM**	SmO_2_-SCM	70	55.69 ± 53.80	0.000 *	
**Upper trapezius**	SmO_2_-UT	70	54.85 ± 3.01	0.010 *	
**Hemoglobin concentration (ThB)**					
**Masseter**	ThB-M	70	12.36 ± 0.41	0.000 *	
**SCM**	ThB-SCM	70	12.40 ± 0.31	0.010 *	
**Upper trapezius**	ThB-UT	70	12.43 ± 0.28	0.078	
**Muscle contraction (Cont)**					
**Masseter**	Cont-M	70	197.15 ± 91.63	0.015 *	
**SCM**	Cont-ECOM	70	60.00 ± 34.26	0.000 *	
**Upper trapezius**	Cont-UT	70	189.80 ± 121.53	0.001 *	

^x^ SD—standard deviation. ^#^ Sig.—statistical significance *p* < 0.05. * Statistical significance.

**Table 3:** Multiple linear regression of the relationship between the quantitative outcomes with respect to sex, measurement period, and age in the study sample.

Outcome	Adjustment	Coef.	SE ^x^	Sig. ^#^ (*p*)	95% CI ^+^		R^2 ^^
**Stress**	Sex	−0.36	1.12	0.747	−2.57	1.85	0.017
	Measurement period	1.66	1.11	0.135	−0.53	3.85	
	Age	−0.02	0.18	0.895	−0.38	0.33	
**Cortisol**	Sex	0.20	0.49	0.685	−0.77	1.17	0.031
	Measurement period	−0.98	0.49	0.046 *	−1.94	−0.02	
	Age	0.01	0.08	0.868	−0.14	0.17	
**Maximum mouth opening**	Sex	−5.46	1.24	0.000 *	−7.92	−2.99	0.207
	Measurement period	0.14	1.23	0.907	−2.29	2.58	
	Age	−0.90	0.20	0.000 *	−1.29	-0.51	
**Oxygen saturation (SmO_2_)**							
**Masseter**	Sex	4.96	1.53	0.002 *	1.93	7.99	0.147
	Measurement period	−3.43	1.52	0.025	−6.43	−0.43	
	Age	−0.58	0.24	0.020 *	−1.06	−0.09	
**SCM**	Sex	−3.16	0.95	0.001 *	−5.04	−1.29	0.137
	Measurement period	3.00	0.96	0.002 *	1.10	4.89	
	Age	0.20	0.15	0.202	−0.11	0.50	
**Upper trapezius**	Sex	3.38	0.77	0.000 *	1.85	4.90	0.225
	Measurement period	−3.47	0.76	0.000 *	−4.98	−1.96	
	Age	0.10	0.12	0.438	−0.15	0.34	
**Myoglobin concentration (ThB)**							
**Masseter**	Sex	0.03	0.07	0.659	−0.11	0.17	0.052
	Measurement period	−0.13	0.07	0.058 *	−0.27	0.00	
	Age	0.02	0.01	0.103	0.00	0.04	
**SCM**	Sex	−0.17	0.05	0.001 *	−0.27	0.09	0.101
	Measurement period	−0.01	0.05	0.842	−0.11	−0.08	
	Age	0.01	0.01	0.178	−0.01	0.03	
**Upper trapezius**	Sex	−0.23	0.04	0.000 *	−0.32	−0.14	0.185
	Measurement period	−0.23	0.04	0.727	−0.07	0.10	
	Age	0.01	0.01	0.319	−0.01	0.02	
**Muscle contraction (Cont)**							
**Masseter**	Measurement period	76.18	11.86	0.000 *	52.72	99.64	0.282
	Sex	−21.24	11.99	0.079	−44.95	2.46	
	Age	5.02	1.91	0.010 *	1.24	8.81	
**SCM**	Measurement period	0.99	5.58	0.859	−10.04	12.03	0.164
	Sex	−27.36	5.64	0.000 *	−38.51	−16.21	
	Age	1.05	0.90	0.246	−0.73	2.83	
**Upper trapezius**	Measurement period	98.08	16.78	0.000 *	64.91	131.26	0.251
	Sex	−44.41	16.95	0.010 *	−77.93	−10.89	
	Age	4.48	2.71	0.100 *	−0.88	9.83	

^x^ SE—standard error. ^#^ Sig.—statistical significance *p* < 0.05. ^+^ CI—confidence interval. ^^^ R^2^—coefficient of determination. * Statistical significance.

**Table 4:** Multinominal logistic regression of the relationship between the *TMJ* status, with respect to sex, measurement period, and age in the students.

Base Outcome: TMJ Status: Not_TMDs	Adjustment	RR ^^^	SE *	Sig. ^#^ (*p*)	95% CI ^+^		Pseudo R^2 ^^
**Mild TMDs**	Sex	2.16	0.86	0.054	0.99	4.73	0.124
	Measurement period	1.96	0.75	0.082	0.92	4.16	
	Age	0.87	0.07	0.067	0.75	1.01	
**Moderate TMDs**	Sex	17.69	12.85	0.000 *	4.26	73.45	
	Measurement period	1.07	0.60	0.902	0.36	3.20	
	Age	1.19	0.10	0.028 *	1.02	1.40	

^^^ RR—relative risk. * SE—standard error. ^#^ Sig.—statistical significance *p* < 0.05. ^+^ CI—confidence interval. ^^^ Pseudo R^2^—pseudo coefficient of determination.

**Table 5:** Multiple linear regression of the relationship between stress and sex, measurement period, and TMJ status in students.

Outcome	Adjustment	Coef.	SE ^x^	Sig. ^#^ (*p*)	95% CI ^+^		R^2 ^^
**Stress**	Sex	−2.10	1.12	0.063	−4.31	0.11	0.137
	Measurement period	1.52	1.05	0.148	−0.55	3.61	
	**TMJ status—No TMDs (base outcome)**						
	Mild TMDs	2.34	1.17	0.047 *	0.03	4.64	
	Moderate TMDs	7.20	1.66	0.000 *	3.92	10.48	

^x^ SE—standard error. ^#^ Sig.—statistical significance *p* < 0.05. ^+^ CI—confidence interval. ^^^ R^2^—coefficient of determination. * Statistical significance.

**Table 6:** Multiple linear regression, stratified by sex, of the relationship between the quantitative outcomes and the measurement period and age in the students.

Outcomes	Sex	Adjustment	Coef.	SE ^x^	Sig. ^#^ (*p*)	95% CI ^+^		R^2 ^^
**Stress**	**M**	Measurement period	2.75	1.87	0.065	−0.18	5.67	0.083
		Age	−0.36	−1.80	0.076	−0.76	0.04	
	**F**	Measurement period	0.25	0.16	0.870	−2.82	3.33	0.133
		Age	1.05	3.05	0.003 *	0.36	1.73	
**Cortisol**	**M**	Measurement period	−1.00	0.61	0.108	−2.22	0.23	0.037
		Age	−0.02	0.08	0.851	−0.18	0.15	
	**F**	Measurement period	−0.95	0.78	0.227	−2.52	0.61	0.030
		Age	0.10	0.17	0.551	−0.24	0.45	
**Maximum mouth opening**	**M**	Measurement period	1.49	1.60	0.355	−1.70	4.68	0.127
		Age	−0.69	0.22	0.002 *	−1.12	−0.25	
	**F**	Measurement period	−1.38	1.87	0.463	−5.11	2.35	0.200
		Age	−1.57	0.42	0.000 *	−2.40	−0.74	
**Oxygen saturation (SmO_2_)**								
**Masseter**	**M**	Measurement period	−0.27	2.48	0.914	−5.22	4.68	0.061
		Age	−0.74	0.34	0.033 *	−1.41	−0.06	
	**F**	Measurement period	−7.24	1.39	0.000 *	−10.01	−4.47	0.310
		Age	−0.07	0.31	0.834	−0.68	0.55	
**SCM**	**M**	Measurement period	−0.28	1.46	0.849	−3.20	2.64	0.021
		Age	0.25	0.20	0.223	−0.15	0.64	
	**F**	Measurement period	−0.95	0.78	0.227	−2.52	0.61	0.420
		Age	0.10	0.17	0.551	−0.24	0.45	
**Upper trapezius**	**M**	Measurement period	−1.12	1.17	0.339	−3.45	1.20	0.016
		Age	0.08	0.16	0.615	−0.24	0.40	
	**F**	Measurement period	−6.26	0.82	0.000 *	−7.91	−4.62	0.488
		Age	0.15	0.18	0.432	−0.22	0.51	
**Myoglobin concentration (ThB)**								
**Masseter**	**M**	Measurement period	0.06	0.11	0.565	−0.16	0.28	0.052
		Age	0.03	0.01	0.061	0.00	0.06	
	**F**	Measurement period	0.00	0.07	0.947	−0.15	0.14	0.012
		Age	−0.01	0.02	0.397	−0.05	0.02	
**SCM**	**M**	Measurement period	0.00	0.07	0.978	−0.14	0.14	0.058
		Age	0.02	0.01	0.038 *	0.00	0.04	
	**F**	Measurement period	−0.02	0.07	0.819	−0.16	0.12	0.021
		Age	−0.02	0.02	0.266	−0.05	0.01	
**Upper trapezius**	**M**	Measurement period	0.02	0.05	0.680	−0.09	0.13	0.060
		Age	0.02	0.01	0.038 *	0.00	0.03	
	**F**	Measurement period	0.01	0.07	0.891	−0.12	0.14	0.030
		Age	−0.02	0.01	0.173	−0.05	0.01	
**Muscle contraction (Cont)**								
**Masseter**	**M**	Measurement period	85.35	19.73	0.000 *	46.03	124.68	0.243
		Age	5.55	2.69	0.043 *	0.19	10.92	
	**F**	Measurement period	65.48	11.32	0.000 *	42.84	88.11	0.370
		Age	3.34	2.52	0.191	−1.71	8.38	
**SCM**	**M**	Measurement period	−10.06	8.58	0.245	−27.16	7.04	0.046
		Age	1.73	1.17	0.144	−0.60	4.06	
	**F**	Measurement period	14.36	6.33	0.027 *	1.70	27.02	0.085
		Age	−1.11	1.41	0.436	−3.93	1.71	
**Upper trapezius**	**M**	Measurement period	92.59	24.14	0.000 *	44.48	140.69	0.241
		Age	9.29	3.29	0.006 *	2.73	15.85	
	**F**	Measurement period	106.30	21.04	0.000 *	64.22	148.38	0.330
		Age	−10.81	4.69	0.025 *	−20.19	−1.43	

^x^ SE—standard error. ^#^ Sig.—statistical significance *p* < 0.05. ^+^ CI—confidence interval. ^^^ R^2^—coefficient of determination. * Statistical significance.

**Table 7:** Multinominal logistic regression, stratified by sex, of the relationship between the *TMJ* status and the measurement period and age in the students.

Base Outcome: TMJ Status: Not TMDs		Adjustment	RR ^^^	SE ^x^	Sig. ^#^ (*p*)	95% CI ^+^		Pseudo R^2 ^^
**Male**	**Mild TMDs**	Measurement period	1.64	0.80	0.313	0.63	4.27	0.052
		Age	0.88	0.07	0.103	0.76	1.03	
	**Moderate TMDs**	Measurement period	1.42	1.53	0.744	0.17	11.75	
		Age	0.52	0.27	0.203	0.19	1.42	
**Female**	**Mild TMDs**	Measurement period	2.30	1.48	0.197	0.65	8.15	0.269
		Age	1.49	0.57	0.303	0.70	3.16	
	**Moderate TMDs**	Measurement period	0.82	0.78	0.838	0.13	5.26	
		Age	3.48	1.44	0.003 *	1.54	7.84	

^^^ RR—relative risk. ^x^ SE—standard error. ^#^ Sig.—statistical significance *p* < 0.05. ^+^ CI—confidence interval. ^^^ Pseudo R^2^—pseudo coefficient of determination. * Statistical significance.

**Table 8:** Multinominal logistic regression, stratified by sex, of the relationship between the *TMJ* status and the measurement period and age in the students.

Outcome	Sex	Adjustment	Coef.	SE ^x^	Sig. ^#^ (*p*)	95% CI ^+^		R^2 ^^
**Stress**	**M**	Measurement period	2.22	1.37	0.107	−0.49	5.94	0.239
		**TMJ status—No TMDs (base outcome)**						
		Mild TMDs	4.37	1.41	0.003 *	1.57	7.18	
		Moderate TMDs	10.39	3.10	0.001 *	4.21	16.56	
	**F**	Measurement period	0.94	1.59	0.556	−2.23	4.11	0.158
		**TMJ status—No TMDs (base outcome)**						
		Mild TMDs	−1.31	1.96	0.506	−5.23	2.61	
		Moderate TMDs	4.10	2.14	0.060	−0.18	8.39	

^x^ SE—standard error. ^#^ Sig.—statistical significance *p* < 0.05. ^+^ CI—confidence interval. ^^^ R2—coefficient of determination. * Statistical significance.

## Data Availability

The raw data supporting the conclusions of this article will be made available by the authors, without undue reservation.
